# Pr^3+^ Visible to Ultraviolet Upconversion for Antimicrobial Applications

**DOI:** 10.3390/nano15070562

**Published:** 2025-04-06

**Authors:** Miroslav D. Dramićanin, Mikhail G. Brik, Željka Antić, Radu Bănică, Cristina Mosoarca, Tatjana Dramićanin, Zoran Ristić, George Daniel Dima, Tom Förster, Markus Suta

**Affiliations:** 1 National Institute of Research and Development for Electrochemistry and Condensed Matter, Str. Dr. A. Păunescu Podeanu nr.144, 300569 Timisoara, Romania; mikhail.brik@ut.ee (M.G.B.); zeljkaa@gmail.com (Ž.A.); radu.banica@incemc.ro (R.B.); cristina.mosoarca@incemc.ro (C.M.); george.dima@upt.ro (G.D.D.); 2 Centre of Excellence for Photoconversion, Vinča Institute of Nuclear Sciences—National Institute of the Republic of Serbia, University of Belgrade, Mike Petrovi12-14, 11000 Belgrade, Serbia; tatjana@vinca.rs (T.D.); risticz@vin.bg.ac.rs (Z.R.); 3 Inorganic Photoactive Materials, Institute of Inorganic and Structural Chemistry, Heinrich Heine University Düsseldorf, Universitätsstraße 1, 40225 Düsseldorf, Germany; tom.foerster@hhu.de (T.F.); markus.suta@hhu.de (M.S.)

**Keywords:** upconversion, lanthanides, Pr^3+^ emission, UVC, antimicrobial effect, germicidal efficiency, antimicrobial photodynamic treatment

## Abstract

This paper addresses the upconversion of blue light to ultraviolet-C (UVC) with Pr^3+^-activated materials for antibacterial applications of UVC. It discusses the processes through which UV radiation provides biocidal effects on microorganisms, along with the most popular UVC sources employed in these processes. We describe the electronic and optical properties of the Pr^3+^ ion, emphasizing the conditions the host material must meet to obtain broad and intense emission in the UVC from parity-allowed transitions from the 4f5d levels and provide a list of materials that fulfill these conditions. This paper also delineates lanthanide-based upconversion, focusing on Pr^3+^ blue to UVC upconversion via the ^3^P_0_ and ^1^D_2_ intermediate states, and suggests routes for improving the quantum efficiency of the process. We review literature related to the use of upconversion materials in antimicrobial photodynamic treatments and for the blue to UVC upconversion germicidal effects. Further, we propose the spectral overlap between the UVC emission of Pr^3+^ materials and the germicidal effectiveness curve as a criterion for assessing the potential of these materials in antimicrobial applications. Finally, this paper briefly assesses the toxicity of materials commonly used in the preparation of upconversion materials.

## 1. Introduction

Recent years have seen increasing adoption of non-thermal and chemical-free disinfection methods, such as ultraviolet (UV)–C light, pulsed UV, cold plasma, and ultrasonic waves. UV radiation effectively combats pathogenic microorganisms by directly interacting with their deoxyribonucleic acid (DNA), leaving no toxic or environmentally stable chemical residues. Since 1877, when the initial scientific report on the germicidal effects of UV radiation was published [1], UV irradiation has been used as an effective antimicrobial strategy in different laboratory and hospital devices, such as UV sterilization lamps and microbiology cabins [2]. It has also been proposed for the treatment of microbial infections in patients [3,4]. Although some microbial species are partially tolerant to UV radiation [3], primarily due to the solar UV levels present in an organism’s natural habitat, it is widely acknowledged that UV radiation adversely affects them [5]. UV radiation can be separated into five wavelength ranges: vacuum-UV (40–190 nm), far-UV (190–220 nm), UVC (220–280 nm), UVB (280–315 nm), and UVA (315–400 nm). The effectiveness of ultraviolet (UV) light in killing germs depends on its wavelength. High-energy radiation (short wavelengths) has a higher potential for damaging microorganisms. However, in 1930, Gates discovered that a characteristic curve of UV radiation bactericidal efficacy has a maximum between 260 and 270 nm [6]. This is a result of specific absorptions of bacterial components that have the largest absorption values around these wavelengths. It should be noted, however, that the wavelength dependence of the UV radiation germicidal effectiveness varies between different species of the same microorganism family, as well as between different types of microorganisms, mostly based on the cell wall structure, DNA arrangement, and repair mechanisms [7], as discussed in more detail in Section 2.

UVC causes the most harm to microorganisms, yet it accounts for only 0.5% of total solar radiation. In fact, ozone in the stratosphere absorbs virtually all the vacuum-UV, far-UV, and UVC; half of the UVB; and some of the UVA radiation before it reaches the Earth’s surface [8]. As a result, we must use artificial UV light sources for disinfecting water, air, and surfaces, as well as for treating tissue infections. The most used sources are discussed in Section 3. A contemporary approach to UV light generation is based on shorter-than-excitation-wavelength (STEW) processes, in which photons emitted by a material have higher energy (shorter wavelengths) than the photons that produced them. In Section 4, we discuss the most widely used STEW process, which is based on lanthanide-facilitated upconversion. Section 5 discusses the application of near-infrared-to-visible lanthanide upconversion nanoparticles in antimicrobial contexts, primarily in antimicrobial photodynamic treatment. This work emphasizes visible-to-UV lanthanide-facilitated upconversion and its germicidal applications (Section 6), primarily focusing on Pr^3+^ blue-to-UVC upconversion. Several reviews published in recent years [9,10,11] on Pr^3+^ blue-to-UVC upconversion signify the importance of this fast-growing research field. Here, we cover the electronic and optical properties of Pr^3+^ ions in Section 7 and explain the mechanisms behind Pr^3+^ blue-to-UVC upconversion in Section 8, along with the analysis of quantum efficiencies of these processes and strategies aimed at improving the efficiency. Section 9 reviews the germicidal applications of UV radiation produced from Pr^3+^ upconversion, highlighting several instances from the literature. The final section briefly discusses the toxicity of the materials frequently used for Pr^3+^ upconversion.

## 2. On UV Radiation’s Germicidal Effects

Microorganism inactivation is facilitated by different processes depending on the UV light wavelength/energy. UVC is a highly effective bactericidal agent due to its strong absorption by DNA (maximum at 265 nm), where it induces the formation of cyclobutane pyrimidine dimers and 6-4 photoproducts, disrupting replication and transcription processes [12]. These photochemical reactions directly damage DNA, leading to cell death. Buonanno et al. [13] recently demonstrated that long-term UVC (222 nm) exposure can efficiently destroy airborne human coronaviruses. Dai et al. [14] demonstrated that UVC was more effective than nystatin cream in lowering the fungal burden of mouse burns with developed infections. UVB operates through similar photochemical reactions, although it is less efficient than UVC because of the small DNA absorption. However, UVB has a greater penetration depth in tissues than UVC, and it reaches greater depths in water, which is important for water disinfection. When used in combination, UVB and UVC show additive effects rather than synergistic inactivation [15]. UVA has a higher tissue penetration depth and much lower DNA absorption than UVC and UVB. Its principal biocidal activity is the production of reactive oxygen species (ROS), which cause oxidative damage to key biological components such as membranes, proteins, and DNA [16,17,18]. This indirect mechanism results in cellular dysfunction and increased vulnerability to subsequent stressors. A particularly effective strategy involves combining UVA pretreatment with UVC irradiation. During UVA exposure, ROS, especially hydroxyl radicals, are generated within bacterial cells, impairing essential functions such as DNA repair mechanisms. This makes the bacteria more susceptible to UVC-induced DNA damage and inhibits their ability to recover through photoreactivation or dark repair [19,20]. For instance, UVA pretreatment can suppress photoreactivation—a process where UV-damaged DNA is enzymatically repaired under visible light—reducing recovery rates from 60% (with UVC alone) to just 15% [19,20]. Applying a fluence of 17 J/cm^2^ UVA resulted in a significant enhancement of UVC inactivation, while 52 J/cm^2^ UVA effectively eliminated the repair shoulder in the fluence–response curve [19]. This enhancement is attributed to oxidative stress caused by UVA, which disables key repair enzymes like photolyase. This combined approach is particularly effective against bacteria like *Escherichia coli*, as they rely on active metabolic pathways and repair systems. However, viruses such as MS2, which lack such systems, show no additional benefit from UVA pretreatment [19,20].

In highly contaminated environments, bacteria are not isolated on surfaces but exist within bacterial biofilms. These biofilms make the germicidal process more challenging. The penetration depth of UVB and UVA radiation into bacterial biofilms is much greater than that of far-UVC light (200–235 nm) due to the lower absorption coefficient of proteins in this spectral range. This makes UVC light with wavelengths greater than 235 nm dangerous for humans, as it can penetrate the natural layer of dead cells on the skin’s surface and potentially cause genetic mutations with carcinogenic potential. In some cases, UVA radiation can temporarily block cell division, though the bacteria remain viable in the long term. Some pathogens can enter a viable but non-culturable (VBNC) state after UV-C treatment, complicating disinfection assessments [21]. Research highlights the phenomenon of photoreactivation, where some bacteria repair DNA damage when exposed to wavelengths over 330 nm [22]. Bacterial species have different sensitivities to UVC radiation. Research by Cairns [23] shows UV doses from 0.4 to 235 mJ/cm^2^ effectively deactivate microorganisms, with the extent of DNA damage correlating directly to UV exposure. Martinez-Hernandez et al. [24] found Salmonella enteritidis highly susceptible to UVC, requiring just 2 mJ/cm^2^ for effective disinfection, compared to higher doses needed for other bacteria. Lim and Harrison [25] confirmed UVC light efficacy (at doses ranging from 0 to 223.1 mJ/cm^2^) in reducing *Salmonella* on green tomatoes, demonstrating effectiveness regardless of the tomato’s orientation. Liu et al. [26] noted that water-assisted UV treatments were particularly effective for decontaminating blueberries contaminated with *Salmonella*, outperforming direct UV exposure methods: 7.9 and 4.6 mW/cm^2^. Sommer et al. [27] found that *E. coli* strains required up to 30 mJ/cm^2^ of UV-C for a 6-log reduction, with strain-specific variability in repair mechanisms. Chun et al. [28] reported that UVC doses between 100 and 800 mJ/cm^2^ reduced *E. coli* O157 counts on fresh salads by 2.16 log CFU/g. Allende et al. [29] demonstrated complete bacterial inhibition on fresh products with UV doses between 3 and 8.5 mJ/cm^2^, though high doses negatively impacted the visual quality of lettuce. Liu et al. [26] highlighted that water-assisted UV treatments improved decontamination rates for blueberries contaminated with E. coli O157 compared to direct UV exposure. Martinez-Hernandez et al. [24] reported that *Listeria monocytogenes* was less sensitive to UVC, requiring 926 mJ/cm^2^ for effective disinfection. Chun et al. [28] observed that UVC doses between 100 and 800 mJ/cm^2^ reduced *Listeria monocytogenes* in fresh salads by 2.57 log CFU/g, showcasing significant inactivation at high doses. Kim et al. [30] explored *Salmonella*, *Typhimurium*, and *Listeria* inactivation on lettuce and found greater reductions when samples were irradiated from both sides and positioned closer to the UV source. Chang et al. [31] have determined the doses of UV light (254 nm) required for a 99.9% inactivation of the cultured vegetative bacteria, total coliforms, and standard plate count microorganisms and found that they were comparable. However, the viruses, the bacterial spores, and the amoebic cysts required about 3 to 4 times, 9 times, and 15 times, respectively, the dose required for *E. coli*. The order of microorganisms’ susceptibility to UV treatment is as follows: fungal spores < bacterial spores < mycobacteria < vegetative bacteria < viruses, according to Gryko et al. [32]. It is important to note here that microorganisms are unlikely to develop resistance to UVC radiation, according to published studies [14,33], despite some assertions to the contrary [34].

**Figure 1:**
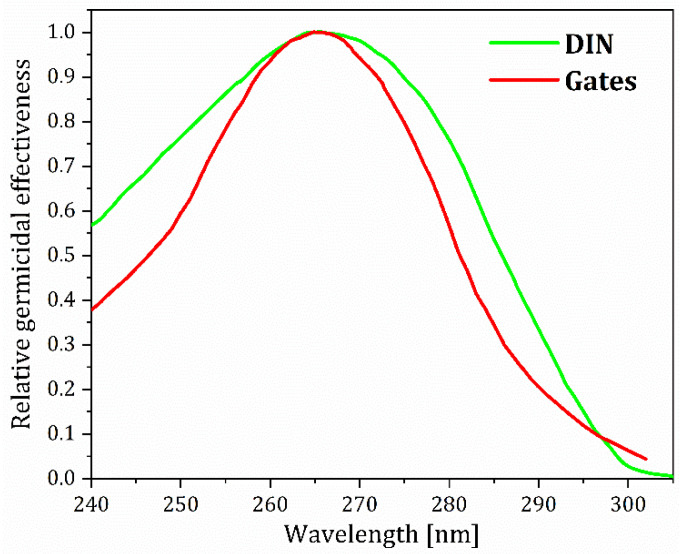
The relative germicidal effectiveness of UV radiation at different wavelengths (the so-called germicidal effectiveness curve—GEC) according to Gates [6] (red line) and DIN standard [35] (green line).

The standard germicidal effectiveness curve shown in Figure 1, which was first created by Gates [6] (red line) and later improved [35] (green line), is widely used to assess the potential of UV sources for germicidal applications. According to these curves, the maximum germicidal efficiency is for the UVC wavelengths in the range from 265 nm to 267.5 nm. If UVC radiation wavelengths are just around 30 nm shorter or longer than the wavelength with the highest efficiency, their effectiveness is reduced to half of their maximum value.

## 3. UVC Light Sources

The sun’s UVC light (<280 nm) is largely absorbed by molecular oxygen and the ozone layer before reaching the Earth [36]. For this reason, antimicrobial applications can only use artificial UVC sources, primarily gas discharge lamps, semiconductor light-emitting diodes (LEDs), and cathodoluminescent light sources (CL); for a review, see Ref. [37], for example.

UV light is often provided from xenon (190–1100 nm), deuterium (190–370  nm), and sealed mercury (253.7  nm) gas discharge lamps [38]. These sources need high operating voltages, are not practical for use because of their large size, and present adverse effects on the environment [38]. Additional drawbacks of current discharge lamps include their fragility, the need for an electronic ballast for operation, mercury toxicity, and the need for specialized materials such as quartz, all of which are currently being addressed by emerging technologies such as pulsed light and dielectric barrier discharge lamps [39]. Low-pressure mercury lamps (with a pressure of ~10 Torr), which account for about 90% of UV disinfection systems and have been used for over 90 years, are highly effective against microorganisms because their monochromatic emission is centered at 253.7 nm, which is very close to the peak of the germicidal efficiency curve (Figure 1). In recent years, these lamps have dramatically reduced mercury consumption, utilizing only 5 mg compared to prior models that used more than 30 mg [40]. Despite having various shortcomings, mercury lamps remain popular in the market owing to their availability. However, due to increased health and environmental concerns about mercury toxicity, the United Nations Minamata Convention [41] is implemented globally to fully ban or considerably decrease the use of mercury gas lamps. Two relatively important innovations are worth mentioning. High-power and high-voltage pulses are used with xenon-filled lamps to generate broad-spectrum white light from which UV can be extracted, while dielectric barrier discharge lamps produce a transient high-pressure glow [42].

The principle of operation of gas lamps containing excimer and exciplex molecules was known beginning in 1970, when the first excimer laser was developed [43]. Because they do not contain mercury, they have become increasingly popular as a replacement for low-pressure mercury lamps [44]. They produce high intensity quasimonochromatic radiation within the range of 100 nm to 350 nm as a result of the excited dimers’ (usually excited by electric discharge) spontaneous decay to their ground state. Different gas mixtures can produce radiation of different wavelengths. For example, the lamp exploiting excimer molecules generates radiation at the following wavelengths: Ar_2_*~146 nm, Kr_2_*~165 nm, and Xe_2_*~172 nm. The radiation wavelengths of lamps that work with exciplex molecules are ArBr*~165 nm, ArCl*~175 nm, KrI*~165 nm, ArF*~193 nm, KrBr*~207 nm, KrCl*~222 nm, KrF*~248 nm, XeI*~253 nm, XeF*~282 nm, and XeCl*~308 nm.

Semiconductors have been used as efficient light-emitting materials for electrically powered light-emitting diodes (LEDs), but the choice of semiconductors for UV light is limited by the necessity for wide band-gap structures (>3.1 eV) that have excellent crystal quality [45]. UVC semiconductor LEDs share the same technology concept as general lightning LEDs, and both are based on the III-nitride materials. However, in order to realize the UV emission, the bandgap of the semiconductor must be increased by substituting In for Al in InGaN (the semiconductor widely used for blue LEDs). The amount of Al required increases as the wavelength of the emission decreases. This presents a considerable technological challenge because Al is a smaller atom than In, and its incorporation causes significant lattice stress and produces structural defects due to its poor match with a crystal lattice. This, combined with challenges in p-type doping, results in relatively low UV LED efficiency [46]. As a result, the cost of producing semiconductor UVC LEDs is significantly higher than that of blue LEDs. On the other hand, UVC LEDs exhibit significant potential for antimicrobial applications due to their compact size and ability to emit at 265 nm (the emission wavelength achieved by optimizing the aluminum content in the semiconductor), where the GEC curve reaches its maximum value. Takano et al. [47] demonstrated an external quantum efficiency (EQE) of 20.3% for an AlGaN-based UVC LED emitting at 275 nm. The EQE reduces below 4% for LEDs emitting at wavelengths shorter than 265 nm owing to a substantial decrease in semiconductor crystal quality.

Cathodoluminescent UVC light sources, also known as electron-beam UVC light sources, have a simpler construction compared to UVC LEDs, with a basic thin-film anode and no p-type layer. An output power of 60 mW has been realized with electron-beam pulse-scanning pumping for a 270 nm emitting device constructed using AlGaN multi-quantum well heterostructures grown on c-Al_2_O_3_ substrates [48]. Kang et al. [49] demonstrated a CL device emitting at 246 nm (FWHM = 16 nm) with an output power of 430 mW via electron-beam pumping of a layer of YPO_4_: Bi^3+^ film. To compete with UVC LEDs, CL sources must be miniaturized in size [50], for example, by using field-emission cold cathodes. Watanabe et al. [51] fabricated a compact device (1.7 × 0.16 cm^2^ emitting surface) using a field-emission array as an excitation source and a hexagonal boron nitride as an emission phosphor. The device has a stable operation output power of 0.2 mW at 225 nm and a quantum efficiency of 0.6% with an excitation voltage of 8 kV. With an anode voltage of 9 kV and a current of 100 mA, the device achieved an output of 1 mW over several hours of operation.

Recent alternatives for creating UVC light sources include the use of non-linear optical (NLO) materials and LEDs, which utilize the blue-to-UVC upconversion from lanthanide-activated phosphors. A detailed discussion of the materials used for the later strategy is given below.

## 4. Short Primer on Lanthanide-Mediated Upconversion

Shorter-than-excitation wavelength (STEW) processes are light-emitting phenomena in which photons released by a material have greater energies (shorter wavelengths) than the original photons that produce them [52]. These processes are a contemporary approach to the production and utilization of light [53]. The primary STEW routes to light conversion are second-harmonic generation and anti-Stokes processes such as two-photon absorption, anti-Stokes Raman, and upconversion (UC). In UC processes, two or more low-energy photons are converted into one high-energy photon, typically through triplet-triplet annihilation and lanthanide-facilitated UC (LnUC), where the former has a better quantum efficiency and is mainly employed for visible-to-visible/UVA UC. LnUC is widely employed in infrared-to-visible UC and, to a lesser extent, visible-to-UVC/UVB/UVA UC [54].

**Figure 2:**
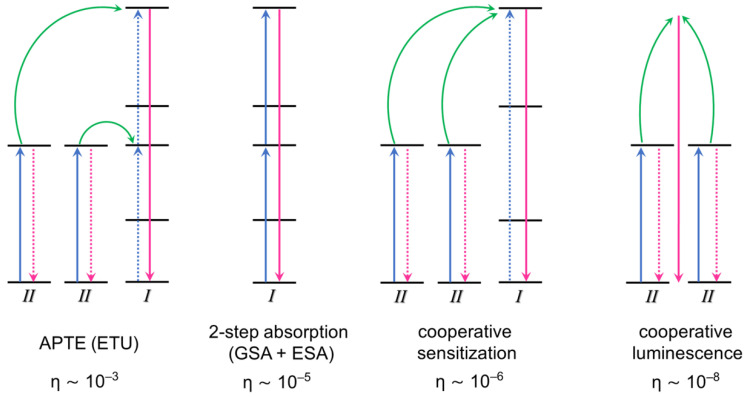
Illustration of 2-photon upconversion mechanisms with their respective quantum efficiencies (η) normalized to the incident power (1 W/cm^2^). Transitions between energy states are indicated by blue and red arrows, while green arrows indicate energy transfer. Adapted from [55], with permission from Elsevier; copyright 1990.

Figure 2 illustrates the common LnUC processes and their efficiencies [55]. According to van der Ende et al. [56], the mechanism known as the APTE effect (Addition de Photon par Transferts d’Energie) [57,58] or ETU (energy transfer upconversion) is the most efficient of all. It may also include ground state absorption (GSA) succeeded by an energy transfer step, frequently denoted as GSA/APTE. The subsequent most effective mechanism is GSA, succeeded by excited state absorption (ESA), in a two-step absorption process. Cooperative sensitization (CS) and cooperative luminescence (CL) mechanisms involve one or more virtual energy levels and are less efficient than the previous two. Other relevant mechanisms are photon avalanche UC, energy migration-mediated UC, and donor–acceptor (D–A) energy transfer (a specific variant of APTE) [52,54,59].

## 5. Antimicrobial Applications of Lanthanide-Facilitated UC

**Figure 3:**
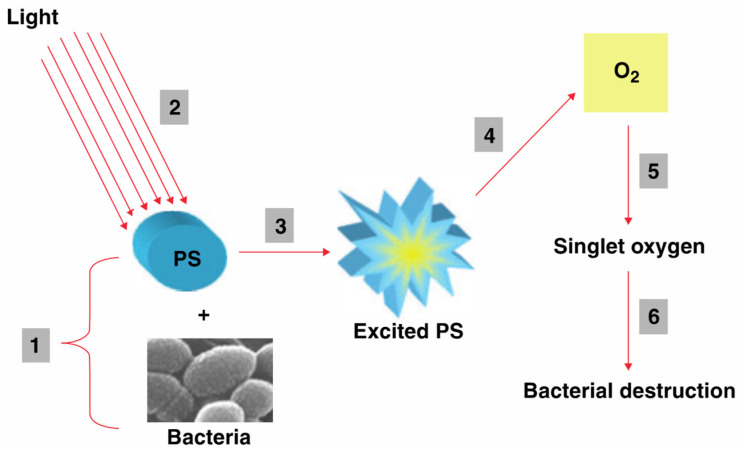
The general principle of antimicrobial photodynamic therapy (aPDT). A photosensitizer is attached to microorganisms (1) and, upon exposure to light of the suitable wavelength (2), becomes energized (3). The photosensitizer subsequently transfers energy to molecular oxygen, resulting in the generation of singlet oxygen and other reactive oxygen species capable of destroying microorganisms. Reprinted from [60] with permission of John Wiley and Sons; copyright 2025.

Because visible light alone cannot effectively destroy microorganisms, near-infrared-to-visible and visible-to-visible UC are primarily used for antimicrobial applications in conjunction with photodynamic treatment (PDT). Antimicrobial photodynamic treatment (aPDT) works on the principle that a photosensitizer, often a photoactive dye, binds to the targeted cells and is triggered by light of a certain wavelength. The photosensitizer then creates reactive oxygen species (ROS), such as singlet oxygen (^1^O_2_), hydroxyl radical (OH^•^), superoxide radical (O_2_^•−^), hydrogen peroxide (H_2_O_2_), etc., causing damage to the microorganism [60]. The first two are the most reactive and most cytotoxic species but have a short diffusion distance [61]. The mechanism underlying PDT antimicrobial action is illustrated in Figure 3 [62]. A photosensitizer is administered and attached to a microorganism, followed by exposure to light that provides suitable energy for its activation. Then, the photosensitizer transfers energy to surrounding oxygen to create ROS that destroy nearby microorganisms.

So far, aPDT has proven to be an effective treatment, in vitro and in vivo, for a wide range of microorganisms, including bacteria, fungi, viruses, and parasites, and is immune to resistance [61,63,64]. However, when aPDT is required to combat infections within tissues, it is impossible to activate photoactive dyes that are responsive to visible light, as tissue strongly absorbs external visible radiation. Considering that these dyes are among the most utilized photosensitizers, it is necessary to find a way to excite them at the location of their action. In contrast to red light, which can penetrate tissues up to 8–10 mm, and green and blue light to even shorter distances, near-infrared radiation at around 980 nm, commonly utilized to excite upconversion materials, achieves penetration depths of up to 10 cm in tissues [65,66]. Therefore, the visible light-responsive photosensitizers can be activated deep in tissues by attaching them to the near-infrared-to-visible UC nanoparticles. Then, near-infrared radiation is converted into visible light by UC nanoparticles, which subsequently activate photosensitizers. UC nanoparticles exploiting Yb^3+^ for near-infrared radiation absorption and Er^3+^ or Tm^3+^ for visible-light emission are typically used for this purpose, as shown in Table 1 [67]. This table also provides compositions of the employed UC nanoparticle-photosensitizer material for UC-facilitated aPDT, the UC host material, dopants involved in UC, absorption and emission wavelengths, and achieved germicidal effect. It is important to note that 980 nm excitation can lead to harmful heating effects because of significant absorption by water molecules, potentially resulting in severe damage to cells and biological tissues. The excitation at 808 nm, where Nd^3+^ absorbs based on its ^4^I_9/2_ → ^4^F_5/2_ transition, presents a promising alternative for near-infrared-to-visible upconversion due to the minimal absorption by water at this wavelength [68].

## 6. Lanthanide-Facilitated Near-Infrared-to-UV(C) and Visible-to-UV(C) UC

Near-infrared-to-UV and visible-to-UV LnUC can be realized with Ce^3+^, Er^3+^, Tm^3+^, and Tm^3+^/Gd^3+^, and Ce^3+^ activators via multiphoton processes (5-photon or more), typically through energy migration [78]/excitation energy-mediated cross-relaxation [79] or with Pr^3+^ through two-photon (visible-to-UV) or three-photon (NIR-to-UV) processes. The latter strategy, Pr^3+^-facilitated UC, is discussed in detail later in the article. The former strategies, which are referred to differently but follow similar principles, require host materials with core–shell or core–multishell morphologies, as well as careful control of dopants in different layers of the structure with high dopant concentrations in some circumstances. However, they are not optimal for antimicrobial applications as they primarily emit in the UVB/UVA spectral regions (except for the weak Gd^3+^ emissions in the 250–315 nm range) and are relatively inefficient due to the requirement of a large number of photons in the conversion process. The typical example of the energy migration UC process (Gd-mediated) is a UVB/UVA emission (300–370 nm) obtained by Yb^3+^ → Tm^3+^ → Gd^3+^ → Ce^3+^ energy transfer in the core–shell–shell nanostructure of NaYbF_4_:Gd/Tm (40/1%)@NaGdF_4_@CaF_2_:Ce (15%) [80].

**Figure 4:**
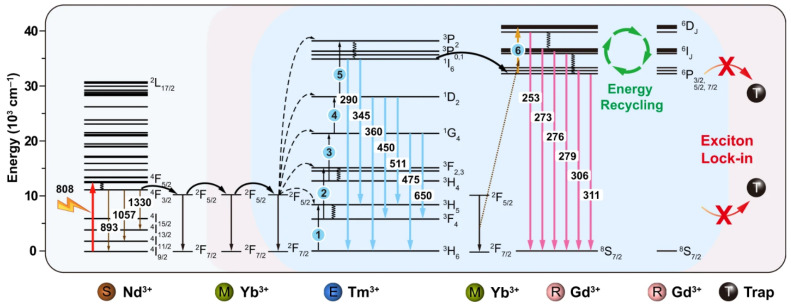
Illustration of the energy transfer sequence Nd^3+^ → Yb^3+^ → Tm^3+^ + Yb^3+^ → Gd^3+^ that enables 808 nm to UVC UC in NaGdF_4_:Yb,Tm@NaGdF_4_:Yb@NaGdF_4_:Yb,Nd@NaGdF_4_ core–multishell nanoparticles. Reprinted from [81] under a Creative Commons Attribution 4.0 international license (Creative Commons CC BY).

Recently, Su et al. [81] demonstrated six-photon UC UV emission from Gd^3+^ (composed of emissions at 253 nm, 273 nm, 276 nm, 279 nm, 306 nm, and 311 nm) under 808 nm excitation using NaGdF_4_:49%Yb,1%Tm@NaGdF_4_:20%Yb@NaGdF_4_:10%Yb,50%Nd@NaGdF_4_ core–multishell nanoparticles. The mechanism is based on the energy transfer sequence Nd^3+^ → Yb^3+^ → Tm^3+^ + Yb^3+^ → Gd^3+^, as depicted in Figure 4. The 808 nm radiation excites Nd^3+^ sensitizers, which pass energy to a lower-energy Yb^3+^ excited state. This is followed by a sequence of five energy transfers from excited Yb^3+^ ions to the ^3^P_2_ state of Tm^3+^ with a subsequent non-radiative relaxation to the Tm^3+ 1^I_6_ level. The energy then passes to the Gd^3+ 6^P_3/2,5/2,7/2_ levels. Finally, energy transfer from Yb^3+^ involves the sixth photon to populate the Gd^3+ 6^D_J_ (J = 9/2, …, 1/2) levels from the ^6^P_3/2,5/2,7/2_ states.

## 7. Electronic and Optical Properties of Pr^3+^ Ions

Praseodymium (Pr) is one of the rare earth elements with atomic number 59. Its complete electron configuration in a neutral state is 1s^2^2s^2^2p^6^3s^2^3p^6^3d^10^4s^2^4p^6^4d^10^5s^2^5p^6^4f^3^6s^2^. In accordance with electron shell filling, the incomplete 4f electron shell—like in the case of other lanthanides—is shielded by the completely filled 5s^2^5p^6^ shells, which considerably decreases the interaction of the 4f electrons with the nearest environment when Pr is incorporated into crystalline matrices. Its most stable oxidation state is +3, and the corresponding electron configuration is [Xe]4f^2^, where [Xe] stands for the electron configuration of xenon.

Various methods of the distribution of two electrons through seven f-orbitals (each orbital is doubly degenerated due to possible orientation of spin) lead to the formation of 91 microstates. Many of those 91 microstates have the same energy and are combined into seven *LS* terms: three spin-triplets, ^3^P, ^3^F, and ^3^H, as well as four spin-singlets, ^1^S, ^1^D, ^1^G, and ^1^I. According to Hund’s rule, the ground term is ^3^H. Spin–orbit coupling is important for the formation of the energy levels of heavy elements (located in the second half of the periodic table) since it is proportional to *Z_eff_*^4^ (*Z_eff_* < *Z*), where *Z* is the atomic number of the element. It splits the *LS* terms into *J*-manifolds, which numbers only 13 in the case of the Pr^3+^ ion: ^3^H_4_, ^3^H_5_, ^3^H_6_, ^3^F_2_, ^3^F_3_, ^3^F_4_, ^3^P_0_, ^3^P_1_,^3^P_2_, ^1^S_0_, ^1^D_2_, ^1^G_4_, and ^1^I_6_. The subscript denotes the values of the total momentum *J*, and in the crystal fields, each of the above-listed states can split into up to 2*J*+1 states, depending on the local symmetry of the crystal lattice site occupied by the Pr^3+^ ions.

**Figure 5:**
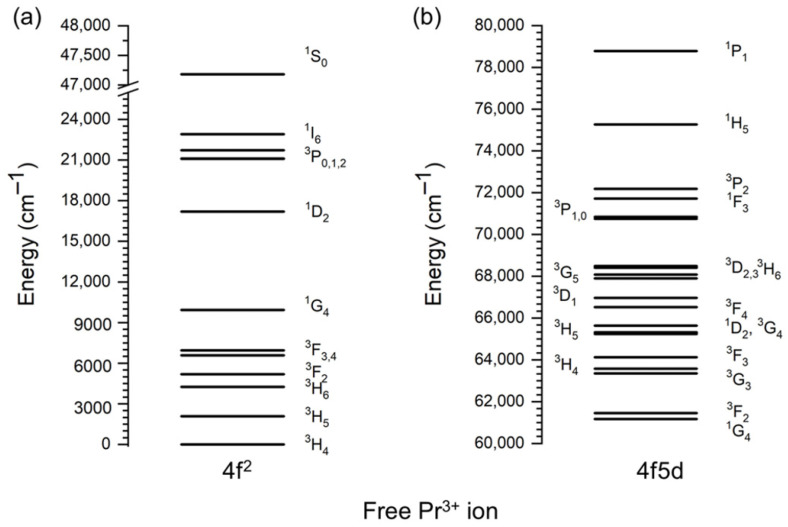
Energy level scheme of a free Pr^3+^ ion for (**a**) 4f^2^ configuration (note the vertical axis break) and (**b**) 4f5d configuration.

Figure 5a shows the energy level scheme of a free Pr^3+^ ion 4f^2^ configuration calculated in Ref. [82] with the free ion Hamiltonian parameters from Ref. [83]. These free ion levels are split in the crystal fields, but the magnitude of such a splitting is not large and does not exceed a few hundred cm^−1^ for each *J*-manifold. This is why the free ion energy level schemes can be used for all lanthanide ions for the analysis and assignment of their spectra in crystals.

The first excited electron configuration of the Pr^3+^ ions is 4f5d, which corresponds to the excitation of one 4f electron into the outer 5d orbitals. This configuration is of the opposite parity to the 4f^2^ states: this is why the 4f–5d excitation (absorption) and 5d–4f (emission) transitions are parity allowed, have high intensity, and appear in the experimental spectra as broad bands. A total of 140 microstates of the 4f5d configuration give rise to 10 *LS* terms: five spin-triplets, ^3^P, ^3^D, ^3^F, ^3^G, and ^3^H, as well as five spin-singlets, ^1^P, ^1^D, ^1^F, ^1^G, and ^1^H. The inclusion of spin–orbit coupling in these states results in the formation of 20 *J*-manifolds: ^3^P_0_, ^3^P_1_, ^3^P_2_, ^3^D_1_, ^3^D_2_, ^3^D_3_, ^3^F_2_, ^3^F_3_, ^3^F_4_, ^3^G_3_, ^3^G_4_, ^3^G_4_, ^3^H_4_, ^3^H_5_, ^3^H_6_, ^1^P_1_, ^1^D_2_, ^1^F_3_, ^1^G_4_, and ^1^H_5_. The lowest excited level of the Pr^3+^ 4f5d configuration is ^1^G_4_ [84]. It is located at 61,171.9 cm^−1^ [84] and is strongly mixed with the ^3^H_4_ state from the same configuration located at 63,580.7 cm^−1^. Although ^3^H_4_ is indicated as the energetically lowest in the seminal work by Crosswhite et al. [85], the wave functions of these states calculated in Ref. [84] are 46% |^1^G_4_> + 49% |^3^H_4_> and 38% |^1^G_4_> + 50% |^3^H_4_> (only two greatest contributions to the wave functions are given, and all the remaining ones are omitted for the sake of brevity). Energy level assignment by the largest component of the wave function indicates that the |^3^H_4_> state produces the largest contribution to wave functions of both states, but since its contribution to the 63,580.7 cm^−1^ level is considerably greater than that of |^1^G_4_>, it is assigned here as the |^3^H_4_> state. Then, the ground state of the 4f5d configuration at 61,171.9 cm^−1^ is assigned to the |^1^G_4_> state; otherwise, two |^3^H_4_> states appear in the energy level scheme, and the |^1^G_4_> state simply vanishes. This is the standard approach to the energy levels assignment in the case of energy levels that are located closely in energy and whose wave functions are strongly mixed. Figure 5b shows the 4f5d levels of the Pr^3+^ ions [86]. The energy levels of the next excited electron configuration, 4f6s, start at around 100,000 cm^−1^.

When the lowest0energy 4f5d state is still higher in energy than the 4f^2 1^S_0_ level, Pr^3+^ ions exhibit several 4f^2^ → 4f^2^ emission transitions, which can be excited in different ways: either by resonant excitation to the emission levels or by non-radiative or down-conversion processes. One of those mechanisms can be UV excitation from the ^3^H_4_ ground state to the ^1^S_0_ level, which is followed by a downward cascade transition to the group of the closely located ^3^P_0_, ^3^P_1_,^3^P_2_, and ^1^I_6_ manifolds following emission transitions to the ground level ^3^H_4_ and nearest excited levels ^3^H_5_, ^3^H_6_, and ^3^F_2_.

**Figure 6:**
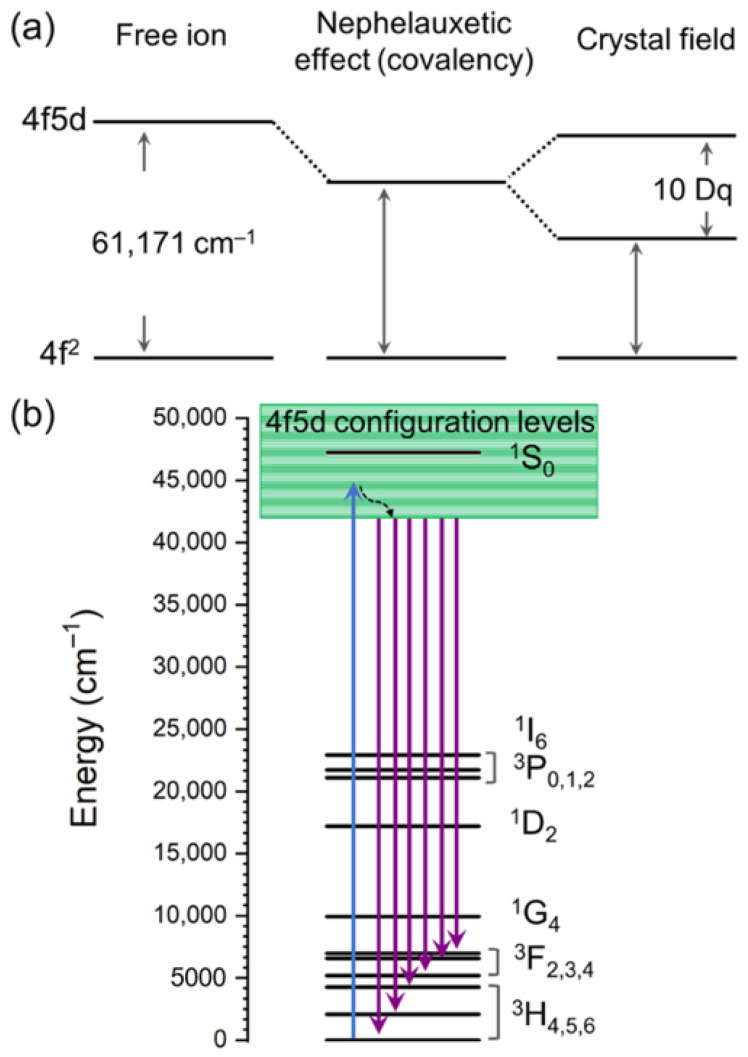
(**a**) Energy gap between 4f^2^ and 4f5d states in Pr^3+^ and (**b**) UV emissions from 4f5d to 4f^2^ transitions.

A particular feature of the Pr^3+^ ions is that the lowest levels of the excited 4f5d electron configuration are located rather close to the ^1^S_0_ level (≈47,200 cm^−1^), which is the highest level of the 4f^2^ electron configuration. According to the NIST Atomic Spectra Database [86], the lowest state of the 4f5d electron configuration is at 61,171 cm^−1^ for the free Pr^3+^ ion, which indicates a strong mixture of states of both 4f^2^ and 4f5d configurations. The situation becomes even more complicated for Pr^3+^ ions in crystalline solids. Since the 5d states are spatially more extended, they interact strongly with the surroundings. In combination with a pronounced nephelauxetic effect (covalency), this can lead to a considerable red shift (decrease in energy) of the 4f5d states (Figure 6a), often even below the ^1^S_0_ level. Such a situation is favorable for obtaining broad UV emission from the Pr^3+^ 4f5d→4f^2^ (Figure 6b) if the Stokes shift of this emission is smaller than 3000 cm^−1^ [87,88].

According to the crystal field theory, the energy of d-orbital splitting depends on the anion charge/anion radius (spectrochemical series): I^−^ < Br^−^ < Cl^−^ < S^2−^ < F^−^ < O^2−^ < N^3−^ < C^4−^, symmetry (coordination number and site symmetry): octahedral > cubic, dodecahedral, square antiprismatic > tetrahedral, and Pr^3+^-ligand distance: Dq $=35Ze/4R^{5}$ (*R* is the cation–anion distance, *Z* represents the valency of anions, and *e* is the electron charge) [89]. The covalency of the Pr^3+^–ligand bonds mainly depends on the polarizability (type) of the anions involved (sulfides > nitrides > oxides > fluorides) and charge density on the surrounding anions (aluminates and gallates > silicates and germanates > borates > phosphates > sulfates) [89]. The increase in the covalency of the Pr^3+^–ligand bonds leads to a decrease in 4f5d energy.

Upon excitation of Pr^3+^ into the 4f5d configuration, a series of emission transitions from that state to the ^3^H_J_ (J = 4, 5, 6) and ^3^F_J_ (J = 2, 3, 4) levels occurs. These transitions are parity allowed, so they provide faster, broader, and significantly more intense emissions (over the 200–400 nm spectral range, depending on the host) compared to the narrow-line emission from parity-forbidden 4f^2^ → 4f^2^ transitions (in the VIS and NIR spectral range). If the lowest energetic 4f5d state and ^1^S_0_ level are close in energy, emissions from both 4f5d → 4f^2^ and 4f^2^ → 4f^2^ transitions occur. Table 2 provides the wavelengths of emissions from the Pr^3+^-based 4f5d spin-allowed transitions in several host materials along with the description of their structure.

Much more comprehensive data can be found in Refs. [90,91]. In instances lacking literature data for the energy of the Pr^3+^ first spin-allowed transition, it can be estimated using an extensive dataset available for Ce^3+^ or from the relatively limited literature data for Nd^3+^, using the following equation [90,91]:

(1) $$∆E\left({Pr}^{3+}\right)\approx ∆E\left({Ce}^{3+}\right)+12,240\;{cm}^{-1}\approx ∆E\left({Nd}^{3+}\right)-10,460\;{cm}^{-1}.$$

## 8. Mechanisms of Visible-to-UV Upconversion in Pr^3+^

Historically, the first UC processes in Pr^3+^ have been demonstrated through observation of blue (^3^P_0_ → ^3^H_4_) and red (^1^D_2_ → ^3^H_4_) emissions under near-infrared excitation (NIR-to-VIS UC) and blue emission under red excitation (VIS-to-VIS UC) [128,129,130,131,132,133,134,135]. The quantum yields of these processes were less than 1% [136,137].

**Figure 7:**
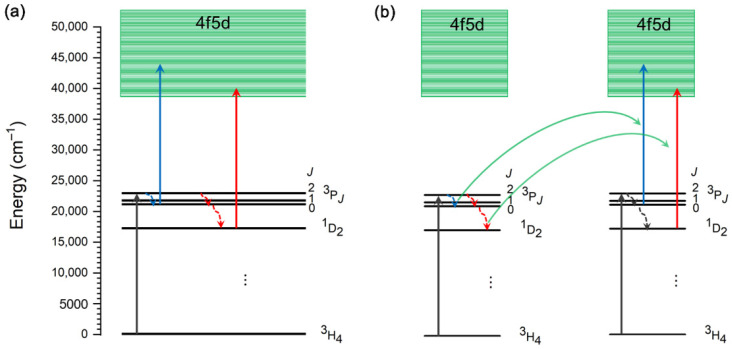
Blue-to-UV UC mechanisms of Pr^3+^ after first excitation with blue light populating the ^3^P_2_ level. (**a**) GSA/ESA process mediated by the ^3^P_0_ (blue arrows) and ^1^D_2_ (red arrows) levels. (**b**) GSA/APTE process mediated by the ^3^P_0_ (blue arrows) and ^1^D_2_ (red arrows) levels.

Upconversion emission from the Pr^3+^ 4f5d state occurs under blue excitation via GSA/ESA (Figure 7a) or GSA/APTE (Figure 7b) processes. Except for low Pr^3+^ concentrations, the former process is more efficient than the latter [138]. Both kinds of processes can take place at the same time, and the formula to determine the ratio of APTE to ESA rates in the UC process was given by Sun et al. [138]. The presence of a delay in the transient of the emitted signal (i.e., the rise in emission before the decay) shows the presence of an energy transfer (APTE), allowing time-resolved emission measurements to be utilized to determine the dominant UC process. Both GSA/ESA and GSA/APTE are two-photon processes and use either ^3^P_0_ (blue arrows) or ^1^D_2_ (red arrows) levels as an intermediate level. For the efficient GSA/ESA UC, a long-lived intermediate state is generally required. Consequently, effective GSA/ESA UC via the ^3^P_0_ level requires hosts with low phonon energies for minimizing non-radiative transitions to the ^1^D_2_ level (^3^P_0_ → ^1^D_2_ multiphonon relaxation and [^3^P_0_,^3^H_4_] → [^1^D_2_,^3^H_6_] cross-relaxation). The ^1^D_2_ level exhibits a significantly longer radiative decay time than ^3^P_0_ since a multiphonon relaxation to the adjacent lower energetic ^1^G_4_ is weak because of a large energy gap of around 6500 cm^−1^ and a weak radiative transition to the ^3^H_4_ ground level (this transition is spin-forbidden). In ^1^D_2_ level-mediated UC, obtaining a strong population of the ^1^D_2_ level through the ^3^P_0_ level is one of the essential conditions to achieve efficient UC, as direct excitation from the ^3^H_4_ to the ^1^D_2_ level is weak. This can be realized in high-energy phonon hosts that promote strong multiphonon relaxation from ^3^P_0_ to ^1^D_2_ or in hosts with a metal-to-metal charge transfer band with energy adequate to bridge ^3^P_0_ to ^1^D_2_ states.

For a free Pr^3+^ ion, the lowest energy 4f5d state is ^1^G_4_ [86]. In Pr^3+^-activated LaCl_3_, as previously stated, the wave function of 4f5d ^1^G_4_ state is strongly mixed by a spin–orbit interaction in fractions of 46% ^1^G_4_ (spin singlet) + 49% ^3^H_4_ (spin-triplet) [84]. The wavefunction of the 4f5d ^3^H_4_ state is also spin–orbit-mixed 50% ^3^H_4_ + 38% ^1^G_4_ [84]; therefore, in both cases, the spin-triplet state dominates, as expected according to Hund’s rules. The degree of mixing between ^1^G_4_ and ^3^H_4_ states may differ across various hosts; however, the fast emission decays and broad emission bands that originate from the lowest energetic 4f5d state to ^3^H_4,5,6_ spin-triplet states indicate the spin-allowed nature of these transitions and the predominant role of the ^3^H_4_ spin-triplet state in the wavefunction of the lowest energy 4f5d state. Therefore, one may argue that both ^3^P_0_ (4f^2^) → 4f5d and ^1^D_2_ (4f^2^) → 4f5d transitions are only partially spin-allowed and that the efficiency of ^1^D_2_-mediated UC depends on the amount of singlet state contribution to the wavefunction of the lowest energy 4f5d state. In this sense, 4f5d → ^3^H_4,5,6_ may also be characterized as partially spin-allowed. It should be noted that for some hosts, UC via the ^1^D_2_ state is not possible if the energy gap between the ^1^D_2_ level and the 4f5d states is greater than the excitation energy to the ^3^P*_J_* states.

The theoretical maximum of UC quantum yield is 50% [139] due to the absorption of two photons per one emitted. However, most of the Pr^3+^ UC materials show significantly smaller UC quantum yields of around 0.01%, which are also substantially lower than the yields of NIR-to-visible lanthanide UCs (quantum yields up to 10% can be achieved in β-NaYF_4_:2% Er^3+^,18% Yb^3+^ [140,141]). For example, previous work [142] indicates a UC quantum yield of 0.0019% (excitation power flux of 1.65 mW cm^−2^) for X2-Y_2_SiO_5_:Pr^3+^,Li^+^, while 3.9-fold stronger UC emission has been observed in β-Y_2_Si_2_O_7_:Pr^3+^ [143]. Recent studies on bromide-based [144] and borate-based materials [145] have demonstrated significantly improved UVC UC. Furthermore, [145] is a good example of a strategy using high-phonon-energy materials to populate the ^1^D_2_ level. UC efficiency is mainly affected by a relatively small absorption cross-section of the ^3^P_0,1,2_ levels [146] and emission quenching due to non-radiative multiphonon relaxations, cross-relaxations, energy transfer/migration processes (such as Pr^3+^–Pr^3+^ interactions), and Pr^3+^ ion interactions with species attached to phosphor particle surfaces [147]. The process of multiphonon relaxation from the ^3^P_0_ level to the ^1^D_2_ level is a key reason why the UC efficiency using ^3^P_0_ as an intermediate level is limited. The energy difference between these two levels (∆E) is around 3800 cm^−1^, so it can be easily bridged by several high-energy phonons. The multiphonon relaxation rate (WMPR) for an excited state resulting from stimulated phonon emission can be derived from the energy-gap law [148]:

(2) $$W_{MPR\;}=\beta \cdot \exp\left[-\alpha \cdot (∆E-2\hbar \omega_{max})\right]\;,$$

where $\hbar \omega_{max}$ is the cutoff phonon energy (maximum optical phonon energy) of the host, and α and β are the characteristic coefficients. For many silicates, which usually have a cutoff phonon energy of about 1080 cm^−1^, it takes only three to four phonons to depopulate the ^3^P_0_ level, with a multiphonon relaxation rate of about 28,500 s^−1^ (the non-radiative lifetime of around 35 μs; calculated using $\alpha =4.7\times 10^{-3}$ s^−1^ and $\beta =9\times 10^{7}$ cm^−1^ [147]). For this reason, high-energy phonon hosts, such as silicates and phosphates (see Table 2), are not well-suited for the UC exploiting ^3^P_0_ as an intermediate level. On the other hand, using high-phonon-energy materials to populate the ^1^D_2_ level has recently been demonstrated as an perspective strategy to increase the efficiency of UC using the ^1^D_2_ intermediate level [145]. At high Pr doping concentrations, usually ≥0.5 mol%, the energy transfer process becomes efficient because of the reduced Pr^3+^–Pr^3+^ distance, and the UC process is hindered by cross-relaxations due to enhanced non-radiative coupling between ions [145,149]. The situation with nanoparticles is somewhat more complicated. Due to phonon confinement, the cutoff phonon energy may be smaller than the maximum optical phonon energy in the bulk. On the other hand, Pr^3+^ ions interact more strongly with species at particle surfaces, for example, OH groups, resulting in the emission quenching due to this interaction. These complex nature of excitation and emission transitions explains the low efficiency of Pr^3+^ two-photon blue-to-UV UC compared to efficiencies of lanthanide-facilitated near-infrared-to-visible UC. However, it is worth noting that substantial research into efficient lanthanide near-infrared-to-visible UC materials has been going on for several decades. Over time, many strategies for increasing UC efficiency have been developed, such as activator concentration optimization; improved material preparation methods, including materials with core/shell morphologies; passivation of particle surfaces; the use of plasmon particles and dyes to enhance absorption, etc. Such efforts have only recently started with Pr^3+^ blue-to-UV UC materials. The Li-codoping of Y_2_SiO_5_:Pr^3+^ resulted in enhanced upconversion emission relative to the non-codoped material, attributed to an increased particle size (flux effect) [126,142]. As the size of crystals enlarge, the number of Pr^3+^ ions in the vicinity of the surface of the particles reduces, thus limiting non-radiative losses [11]. Malyukin et al. [150] suggested that Li^+^-codoping of Y_2_SiO_5_:Pr^3+^ inhibits the clustering of Pr^3+^ ions within the host, hence reducing unwanted cross-relaxation depopulation of the ^1^D_2_ level. However, the mechanism responsible for the improvement in UC efficiency remains ambiguous according to Cates et al. [126]. Apparent strategies for the improvement in Pr^3+^ blue-to-UV UC can be the coupling of the excitation process with the localized surface plasmon resonance (LSPR) of noble metals or the implementation of dye sensitization to enhance the absorption efficiency of UC materials, as molecular dyes possess significantly greater absorption cross-sections (10^−17^–10^−16^ cm^2^) in contrast to the 4f^n^-4f^n^ absorptions of lanthanides (<10^−20^ cm^2^) [151], similarly to the strategies employed with the NIR-to-VIS UC materials [59]. However, these additive materials show high UV absorption, implying that the resultant UC will be absorbed by them. UC efficiency may be improved by coupling the emission with LSPR of UV plasmonic metals, such as Al, Ga, and Rh, but this is yet to be tested.

## 9. Antimicrobial Applications of Lanthanide-Facilitated Visible-to-UVC UC

**Figure 8:**
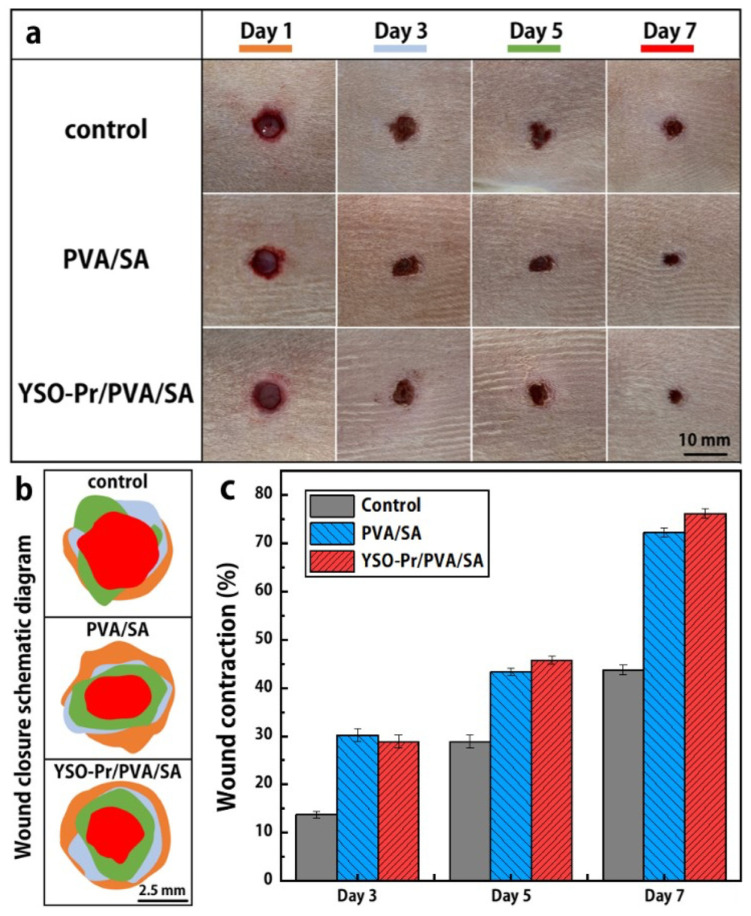
(**a**) Assessment of the biocidal effectiveness of polyvinyl alcohol/sodium alginate and Y_2_SiO_5_:Pr^3+^/polyvinyl alcohol/sodium alginate simulating *S. aureus* infection in vivo. (**b**) Illustration of the wound closure and (**c**) wound contraction rate on the 3rd, 5th, and 7th day. Reprinted from [152] with permission of the Royal Society of Chemistry; copyright 2023.

Table 3 shows a selection of materials doped with Pr^3+^ that have been used to generate UVC UC emission that resulted in a biocidal effect. Zhao et al. [152] conducted in vivo animal studies in addition to the standard practice of demonstrating the germicidal effects of Pr^3+^ UVC emission by exhibiting the findings of in vitro antibacterial research. The authors developed the antibacterial wound dressing in the form of a polymeric antibacterial composite film that is composed of polyvinyl alcohol, sodium alginate, and Y_2_SiO_5_: Pr^3+^. The film was successfully used to inhibit bacteria in actual wounds, as illustrated in Figure 8.

**Figure 9:**
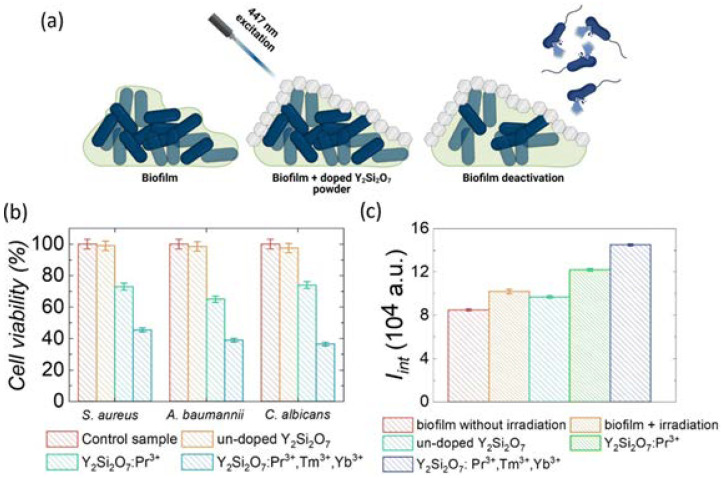
(**a**) An illustration of the proof-of-concept biofilm deactivation with Pr^3+^-doped Y_2_Si_2_O_7_ and Pr^3+^,Yb^3+^,Tm^3+^-doped powders. (**b**) Viability of biofilm (*S. aureus*; *A. baumannii*, and *C. albicans*) cells after irradiation with 447 nm laser light (800 mW/cm^2^) in the presence of un-doped Y_2_Si_2_O_7_; Y_2_Si_2_O_7_:Pr^3+^, and Y_2_Si_2_O_7_:Pr^3+^,Tm^3+^,Yb^3+^ and without them (control, 100% represents viability of non-irradiated cells). (**c**) Effectiveness of generating ROS in biofilm irradiated by a 447 nm laser diode (800 mW/cm^2^) in the presence of un-doped Y_2_Si_2_O_7_; Y_2_Si_2_O_7_:Pr^3+^; and Y_2_Si_2_O_7_:Pr^3+^,Tm^3+^,Yb^3+^ phosphors. Reprinted from [157] under a Creative Commons Attribution 3.0 international license (Creative Commons CC BY).

Falat et al. [157] conducted Tm^3+^ co-activation of Pr^3+^-activated Y_2_Si_2_O_7_ powders to achieve a 370 nm UVA up-conversion emission (Tm^3+^), alongside Pr^3+^ emissions in the UVC (278 nm) and UVB (308 nm) spectrum regions. The authors evaluated the biocidal efficacy of materials subjected to 447 nm laser irradiation on biofilms formed by *A. baumannii*, *S. aureus*, and *C. albicans* (Figure 9a). They discovered that the application of Y_2_Si_2_O_7_:Pr^3+^,Tm^3+^,Yb^3+^ powder as a UV light source resulted in a reduction in biofilm-forming microorganism viability to 45.5 ± 2.5% (*S. aureus*), 39.0 ± 3.0% (*A. baumannii*), and 36.5 ± 2.5% (*C. albicans*), significantly surpassing the results obtained with sole Pr^3+^ doping (approximately 25–35%), as illustrated in Figure 9b). The authors observed an elevation in the concentration of reactive oxygen species (ROS) following 10 min of irradiation of the biofilm, in contrast to the control sample (biofilm without irradiation), as depicted in Figure 9c). The observed increase in the ROS concentration (~54 ± 2%) in Y_2_Si_2_O_7_:Pr^3+^ powders led the research group to conclude that the phototoxicity mechanism of only Pr^3+^-doped materials include both DNA damage and ROS production.

Recent reports indicate that Pr^3+^ UVC emissions obtained through upconversion can be effectively employed for a range of important applications in addition to antimicrobial use. For example, Li_2_SrGeO_4_:Pr^3+^ is used for UVC optical marking, including static and dynamic labeling [159], and the upconversion emission of YOBr:Pr^3+^/polydimethylsiloxane is employed to induce photocatalytic water splitting via NiO-loaded NaTaO_3_:La [144].

The emission from Pr^3+^ does not necessarily need to be produced through upconversion for it to be applicable in sterilization applications. Zhang et al. [160] demonstrated 24 h of continuous 222 nm UVC persistent luminescence in X-ray excited Sr_2_P_2_O_7_:Pr^3+^ phosphor. This phosphor was able to effectively inactivate infectious methicillin-resistant *Staphylococcus aureus* (MRSA) within 30 min in an excitation-free manner. Cheng et al. [161] demonstrated that the inactivation of *Staphylococcus aureus* bacteria under 254 nm mercury lamp irradiation is substantially enhanced when the bacteria are irradiated through PDMS film containing 265 nm-emitting Ba_2_MgSi_2_O_7_:0.4%Pr^3+^ phosphor particles.

**Figure 10:**
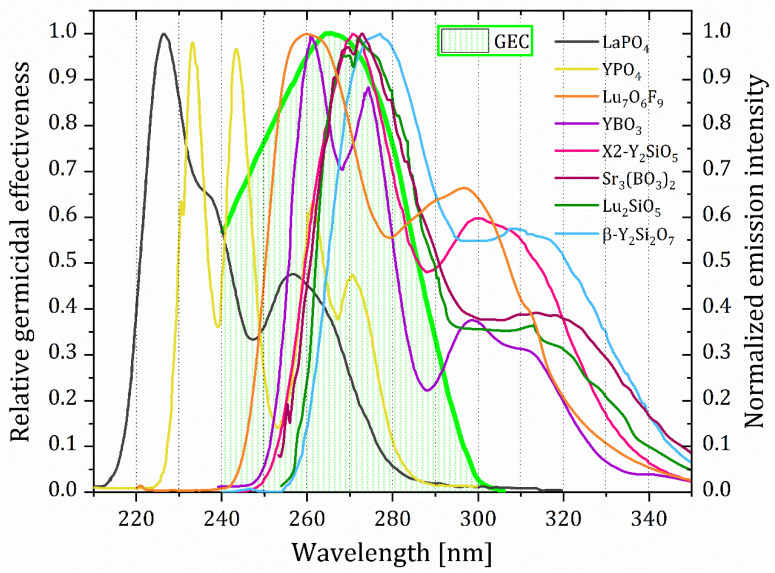
The relative germicidal efficiency curve (green-shaded spectral region; according to DIN [35]) and 4f5d UC emission spectra overlap in certain Pr^3+^-activated hosts often used in Pr^3+^-based blue-to-UVC UC.

It is important to point to the spectral overlap between 4f5d emission spectra with the germicidal efficiency curve (GEC), Figure 10, as the criteria for assessing the potential of the Pr^3+^ UC material for antimicrobial use in addition to its quantum efficiency. Figure 10 shows that only a fraction of the LaPO_4_:Pr^3+^ 4f5d-based emission (28.2%) falls into the GEC spectral region (green-shaded), whereas much of the Lu_7_O_6_F_9_:Pr^3+^ 4f5d-based emission (67.4%) falls in the GEC region with the highest efficiency.

Here, we propose a spectral overlap coefficient (SOC) as one of the key indicators of the Pr^3+^ UVC-emitting materials’ germicidal potential in the following form:

(3) $$\mathrm{SOC}=\frac{\int_{\lambda_{min}}^{\lambda_{max}}GEC\left(\lambda \right)\cdot I_{n}\left(\lambda \right)\cdot \;d\lambda }{\int_{\lambda_{min}}^{\lambda_{max}}GEC\left(\lambda \right)\cdot d\lambda },$$

where λ represents the wavelength, $I_{n}\left(\lambda \right)$ is the normalized emission intensity of the material (normalized to the maximum intensity value of 1), and $GEC\left(\lambda \right)$ stands for the germicidal efficiency curve. Table 4 provides the SOC values for Pr^3+^ materials that are displayed in Figure 10. The value of germicidal potential (GP) of the material can be used for comparison between different Pr^3+^ activated materials:

(4) GP=SOC×EQE,  oralternatively     GP=SOC×Emissionpower fluxExcitationpowerflux,

where EQE is the external quantum efficiency of the material. It is important, however, to remember that GEC is mainly derived from the UV light germicidal effect on *E. colli* bacteria, so it can take different values and spectral shapes for different microorganisms, as discussed in Section 2. Furthermore, the GEC does not cover wavelengths below 240 nm, where a further decrease its value is not necessarily guaranteed to happen. While deriving his germicidal effectiveness curve, Gates [6] found that the germicidal effect on *E. colli* starts to increase with a decrease in the UV light wavelength below 240 nm. It is therefore important to derive the GEC curve for wavelength values less than 240 nm in the future. Additionally, we wish to highlight that GEC can vary depending on the specific microorganism, as noted in Section 2. Therefore, the SOC needs to be adjusted for other pathogens, such as *C. albicans* or MRSA, by deriving and utilizing microorganism-specific GECs.

## 10. The Toxicity of Some Elements Frequently Used for Lanthanide Upconversion Materials

The toxicity of elements commonly employed in lanthanide UC materials raises serious concerns about their environmental and health effects [162,163]. Like other chemicals or pharmaceuticals, properties that are advantageous from optical and biomedical standpoints can also lead to unexpected potentially hazardous toxicities [164]. The issue is particularly significant with nanoparticles [165], which exhibit greater toxicity to human health compared to larger particles of the same chemical substance; it is commonly proposed that toxicity levels are inversely related to the size of the nanoparticles [166,167,168]. As researchers look for safer alternatives, thorough investigations of these materials’ biocompatibility and ecological footprint become increasingly important. Moreover, it is critical to raise awareness of the potential risks associated with the preparation and use of these products.

### 10.1. Yttrium

Occupational exposure to yttrium has been documented in workers involved in electronic waste recycling. These individuals exhibit increased levels of yttrium in their blood and urine compared to the general population. In one study, Y levels were reported at 10–15 μg/L in urine, significantly higher than those observed in the general population (5 μg/L) [169]. This suggests the accumulation of this element in the body due to repeated exposure. Yttrium oxide (Y_2_O_3_) has been shown to induce apoptosis and necrosis in HEK293 cells (human embryonic kidney cells) through mechanisms involving elevated levels of reactive oxygen species (ROS) and disruption of mitochondrial function [170]. This element affects the integrity of the mitochondrial membrane and causes oxidative damage to DNA. In human endothelial cells exposed to Y_2_O_3_ nanoparticles (concentrations of 10 μg/mL), a 40% increase in IL-8 and ICAM-1 levels was observed compared to the control group [171]. This may contribute to the development of chronic diseases, especially in cases of long-term exposure [171]. Liu et al. [172] mention an association between yttrium exposure and hormonal level changes, such as a 20% decrease in TSH levels in infants exposed during pregnancy, indicating endocrine system disruption. In a study on smokers, yttrium concentrations in sperm samples were reported at 1.5–2 μg/L, significantly higher than those detected in non-smokers (approximately 0.8 μg/L). Prolonged exposure was correlated with increased DNA fragmentation in sperm and reduced sperm motility [172]. Prolonged exposure to yttrium in rats causes testicular damage, reducing sperm quality and serum testosterone while increasing cellular apoptosis and cytosolic Ca^2+^ levels [173].

In Y_2_SiO_5_-type compounds, it is likely that, in biological systems, due to the complexation of Y^3+^ with amino acids containing COO^−^ groups and the exposure of particles in the acidic environment of the stomach, their surface becomes enriched in silicon. This may reduce further solubilization of the particles, decreasing the toxicity of Y^3+^ and the rare earth elements with which the compound is doped. The same does not apply to fluorides, which can solubilize to a much greater extent in the human digestive system.

### 10.2. Gadolinium

The toxicity of gadolinium is well documented, as it is administered as a contrast agent for MRI. It has been observed that Gd can remain in bone and brain tissues for up to 8 years after exposure [174]. Gadolinium-based contrast agents (GBCA) can induce acute kidney injury, especially in patients with chronic kidney disease. In patients with diabetic nephropathy, the risk of acute kidney injury increased by 60% after the use of GBCA [175]. Nephrogenic systemic fibrosis (NSF) has been reported as a severe adverse reaction in patients with stage 5 chronic kidney disease. Studies have indicated that gadolinium administered in contrast agents, such as gadodiamide, can trigger this condition [176,177]. Gadolinium oxide nanoparticles (Gd_2_O_3_) induced cytotoxicity in human umbilical vein endothelial cells (HUVEC). At a concentration of 50 μg/mL, Gd_2_O_3_ caused lipid peroxidation and increased ROS, mitochondrial dysfunction, and apoptosis. This underscores the high toxicity of Gd at elevated concentrations [178]. GBCAs have been reported to induce chronic pain, including fibromyalgia, after repeated administrations of Gadovist, a GBCA agent [179]. In other studies, Omniscan (another GBCA) increased the levels of matrix metalloproteinase-1 (MMP-1) and the tissue inhibitor of metalloproteinase-1 (TIMP-1) in human dermal fibroblasts [180]. In vitro studies have shown that gadolinium can stimulate fibroblast proliferation and increase hyaluronic acid production without affecting collagen synthesis. This suggests that Gd may contribute to fibro-trophic processes in tissues [181].

### 10.3. Erbium

In industries using erbium (e.g., manufacturing optical components), workers may be exposed to elevated levels of dust containing erbium. In one study, erbium levels in industrial dust were estimated at 3–5 mg/m^3^, and prolonged exposure was associated with respiratory irritation and chronic inflammation [182]. Erbium-doped nanoparticles induced apoptosis and necrosis in human bronchial epithelial cells (BEAS-2B). Studies showed that YAl_3_(BO_3_)_4_ (YAB) nanoparticles doped with erbium increased ROS levels and affected mitochondrial integrity at concentrations of 20–50 μg/mL [182,183]. In vitro studies indicated a decrease in cell viability by over 30% following prolonged exposure (48–72 h) to YAB nanoparticles doped with erbium. These effects were more pronounced in pulmonary cells compared to other cell lines. Erbium-based materials used in dentistry have not shown significant cytotoxic effects on healthy tissues in routine clinical applications. However, studies recommend avoiding repeated exposure in industrial environments [184].

### 10.4. Lutetium

Lutetium-177 (^177^Lu) is used in radiotherapeutic treatments for cancer, particularly in peptide receptor radionuclide therapy (PRRT). In these applications, toxicity is primarily associated with the emitted radiation. The most common adverse effects include myelosuppression (a reduction in blood cell counts) and renal toxicity, observed in patients exposed to doses of 5.55 GBq (^177^Lu) per treatment [185]. Lutetium is reported to be relatively chemically inert in its non-radioactive forms; however, studies have shown that lutetium-doped nanoparticles can provoke oxidative effects in exposed cells. For instance, lutetium-doped borate nanoparticles induced a decrease in the viability of human lung cells (A549) by approximately 20% at a concentration of 50 μg/mL through oxidative mechanisms [183]. In preclinical studies, lutetium-doped nanoparticles used in radiotherapeutic therapies demonstrated a low toxicity profile in normal cells. However, their chronic use requires further studies to assess long-term risks [184]. Workers in industries handling lutetium, such as laser manufacturing or materials for radiotherapy, may be exposed to aerosols or dust containing this element. Compared to La, the lightest lanthanide, Lu is 200 times more toxic [186]; studies have reported lutetium concentrations of up to 2–3 mg/m^3^ in these environments, but the direct toxic effects on health have not been well-documented [187].

### 10.5. Thulium

Thulium (Tm) is one of the least studied rare earth elements. The available literature identifies health effects primarily related to its nanotechnological and medical applications. Tm^3+^-activated nanoparticles have been used in radiotherapy to enhance the efficiency of treatments against metastases. In industrial settings, repeated exposure to Tm can occur through the handling of materials containing this element. Reported levels in industrial air range between 1 and 3 mg/m^3^. Direct health effects on workers have not been detailed, but it is considered that Tm, similar to other rare earth elements, may contribute to respiratory irritation and chronic inflammation with prolonged exposure [188]. Adverse effects include oxidative stress and changes in gene expression associated with apoptosis, but only at high doses. In therapeutic applications, Tm-activated nanoparticles have proven effective and relatively safe for healthy tissues [189]. Studies have shown that thulium nanoparticles can induce apoptosis in cancer cells, such as cutaneous squamous cell carcinoma, at concentrations of 50 μg/mL. In normal cells, cytotoxicity was minimal, suggesting a promising potential for selective therapeutic treatments [189]. At high exposures, Tm^3+^-activated nanoparticles can cause oxidative stress by increasing ROS levels. In experiments on bronchial epithelial cells (BEAS-2B), Tm^3+^-activated nanoparticles reduced cell viability by 15–20% within 24 h, suggesting oxidative effects at prolonged exposures [190].

### 10.6. Praseodymium

Praseodymium (Pr) is used in various technological applications, including alloys, lasers, and optical materials. Studies on its toxicity have highlighted oxidative effects and its cytotoxic potential, particularly in cases of occupational exposure and contaminated environments. Praseodymium, similar to other rare metals, exhibits low to moderate toxicity. The ingestion of soluble praseodymium salts poses mild toxicity, whereas insoluble salts are non-toxic. These substances are irritants to the skin and eyes. Praseodymium poses significant hazards in occupational settings, primarily because its dust and gases can be inhaled.

Praseodymium nanoparticles (Pr_2_O_3_) have demonstrated significant cytotoxicity in human lung cells (A549). Research has shown a reduction in cell viability by approximately 25% at a concentration of 50 μg/mL due to increased ROS levels and mitochondrial dysfunction [185]. In vitro experiments revealed that Pr caused oxidative DNA damage in bronchial epithelial cells, contributing to apoptosis through the generation of reactive oxygen species. These effects were more pronounced with prolonged exposure, exceeding 24 h, to Pr_2_O_3_ nanoparticles [191,192].

Workers in industries involved in electronic waste recycling or alloy manufacturing containing praseodymium are exposed to dust or aerosols containing this element. Reported levels in industrial air range from 2 to 4 mg/m^3^, and chronic exposure has been associated with respiratory symptoms such as chronic irritation and inflammation [193] Experimental studies suggest that the accumulation of praseodymium in the body can affect the central nervous system by disrupting normal neuronal functions, although the exact mechanisms remain unclear. In animal models, accumulation in the cerebral cortex has been linked to increased oxidative stress [194].

Pr is also used in pesticides and fertilizers, leading to soil and water contamination. Exposure through the food chain can result in accumulation in the human body, with some studies suggesting it may contribute to reduced total protein and serum albumin levels in exposed populations [195].

In biological experiments utilizing UPM doped with Pr^3+^, it is crucial to consider the toxicity of the elements on cell lines to prevent the potential synergistic effects of metal cation toxicity and ultraviolet light emitted by the material. Pr exhibits notable antibacterial activity by disrupting bacterial membrane integrity and permeability, thereby inhibiting bacterial survival rates. The primary mechanism involves ion exchange and the production of reactive oxygen species (ROS), which collectively impair normal cellular functions. The interaction of praseodymium ions (Pr^3+^) with bacterial membranes results in structural destabilization and increased permeability. Studies on *Escherichia coli* have demonstrated that Pr^3+^ displaces calcium ions (Ca^2+^) from their membrane binding sites due to similar ionic radii, thereby exacerbating membrane instability and promoting ion leakage [196,197]. Pr^3+^ ions induce ROS generation within bacterial cells, leading to oxidative stress and eventual cell death. Research on aquatic microorganisms, including *Vibrio fischeri* and *Tetrahymena thermophila*, highlights Pr^3+^ toxicity across a range of effective concentrations (3.5–21 mg/L), with cell death being directly linked to oxidative damage [198].

### 10.7. The Toxicity of Fluorides

Fluoride is a chemical ion naturally present in water, soil, food, and dental hygiene products, such as toothpaste. While fluoride has well-known benefits in preventing dental cavities, excessive exposure can have toxic effects on human health. The acute toxicity of fluoride is due to its ability to form complexes with Ca ions in the body, leading to severe hypocalcemia, which can impair the normal functioning of the nervous and muscular systems [199]. Acute exposure to high concentrations of fluoride, either through accidental or intentional ingestion, can cause nausea, vomiting, abdominal pain, diarrhea, and, in severe cases, respiratory or cardiac failure. The median lethal dose (LD50) for sodium fluoride is approximately 5 mg/kg body weight for an adult [199]. Chronic exposure to excess fluoride (concentrations above 1.5 ppm in drinking water) can lead to dental fluorosis [200]. Prolonged consumption of water with high fluoride levels (over 4 ppm) can cause skeletal fluorosis, characterized by the accumulation of fluoride in bones, making them denser but more brittle [201]. Some epidemiological studies suggest that chronic fluoride exposure may impact cognitive development. A study in China found that children from areas with high fluoride levels in water had lower IQ scores compared to those from areas with normal levels [202]. Fluoride interferes with thyroid function by inhibiting the conversion of T4 to T3, which can lead to subclinical hypothyroidism. This effect has been observed particularly in populations exposed to high fluoride levels [203]. Long-term exposure to high doses of fluoride can affect kidney function, especially in individuals with chronic kidney disease. Fluoride can reduce the ability of kidneys to excrete this ion, leading to its accumulation in the body [204]. Other alkali metal ions, such as Cs^+^, Li^+^, and Na^+^, do not exhibit significant toxic effects at the low concentrations that may arise in industrial applications using UC materials. However, the presence of these elements in chemical compounds is associated with high solubility, necessitating the integration of such materials into polymer composites, microencapsulation, or coatings of particles with other low-solubility inorganic materials.

## 11. Conclusions

Because of its unique features, UVC radiation has found important and contemporary applications, for example, in the decontamination and disinfection of environments, water, and food; in cancer therapy; in photocatalysis; and for invisible identification tags. Some Pr^3+^-activated materials can generate intense UVC light from their excited 4f5d states through mechanisms of scintillation, downshifting, cathodoluminescence, and upconversion, provided that the lowest energetic 4f5d state has a lower energy than the ^1^S_0_ level of the 4f^2^ electron configuration. When excited by blue light into the ^3^P*_J_* levels, Pr^3+^ can transfer the energy of two blue photons into one UVC photon via the GSA+ESA or GSA+ETU process, using ^3^P_0_ or ^1^D_2_ level as an intermediate state. It is not uncommon for all processes to take place concurrently. Blue-to-UVC UC opens up the opportunity for utilizing easily available and inexpensive blue light sources for the aforementioned applications. Although rather inefficient, with values of quantum yields well below 1%, Pr^3+^ blue-to-UVC UC materials have already demonstrated exceptional antimicrobial efficiency, as reported in a number of publications. Their biocidal efficacy can be enhanced by coupling them with inorganic photocatalysts, such as ZnO, TiO_2_, and BiOCl, for antimicrobial photodynamic treatment, analogous to the application of near-infrared-to-visible upconverters with visible-excitable photosensitizers. The alternative approach for UVC generation by lanthanide upconversion materials is mainly via Yb^3+^ to Tm^3+^ energy transfer-mediated upconversion, which is highly inefficient since it requires five near-infrared photons to generate one UVC photon. The low upconversion efficiency of Pr^3+^ upconversion materials is a consequence, however, of much less work devoted to the development of these materials compared to work committed to the development of near-infrared-to-visible upconverters. The UVC generation efficiency can be improved by (1) careful selection of host materials for Pr^3+^ that considers the matching of excitation energy with energies of ^3^H_4_ → ^3^P_J_ absorptions and appropriate phonon spectrum, (2) improving synthesis methods to obtain well-crystalline material, and (3) using charge compensation where appropriate. To enhance the germicidal efficiency of the material, it is crucial to achieve a substantial overlap between the 4f5d emission spectrum and the germicidal efficiency curve alongside the enhancement of upconversion quantum efficiency. This can also be accomplished by selecting appropriate hosts for Pr^3+^. Finally, it is essential to raise awareness regarding the toxicity of materials used in the production of upconversion materials, especially nanoparticles, when real-case applications are considered.

## Figures and Tables

**Table 1:** Some examples of antimicrobial photodynamic therapy (aPDT) applications, including the materials used, the UC hosts, the dopants that play a role in UC, the absorption and emission wavelengths, the germicidal effect that was achieved, and a literature reference.

Material	UC Host Doping Ions (Concentrations)	Excitation and Emission	Antimicrobial Effect	Ref.
CuS-decorated NaYF_4_ nanoparticles coated with methylene blue doped silica and grafted with chitosan	NaYF_4_ Mn^2+^/Yb^3+^/Er^3+^ (30/18/2 mol%)	Exc. 980 nm emission in red (651 nm)	Synergistic photothermal and photodynamic therapy effective against Gram-positive *S. aureus* and Gram-negative *E. coli.*	[69]
NaYF_4_@mSiO_2_ mSiO_2_ (mesoporous silica) shell loaded with hydrophobic photosensitizer SiPc (silicon 2,9,16,23-tetra-tert-butyl-29H,31H-phthalocyanine dihydroxide)	Cubic NaYF_4_ Yb^3+^/Er^3+^ (20/2 mol%)	Exc. 976 (power density of 2 W/cm^2^) emission in green (520–560 nm) and red (640–680 nm)	Complete eradication of *E. coli* and seven-order-of-magnitude decrease in colony-forming units of *S. aureus*.	[70]
Roussin’s black salt (RBS)-loaded UCNPs NaGdF_4_@mSiO_2_@qC (qC—quaternized ammonium chitosan)	NaGdF_4_ Yb^3+^/Tm^3+^ (25/0.3 mol%)	Exc. 980 nm (1 W) emission in UV (290, 345, and 362 nm), blue (450 nm and 474 nm), red (574 nm and 643 nm), and near-infrared (807 nm)	Nitric oxide triggered antibacterial activity against methicillin-resistant *S. aureus* (MRSA) and *E. coli* in vitro and in vivo.	[71]
*N*-octyl chitosan-coated NaYF_4_:Yb,Er@NaYF_4_ core–shell nanoparticles loaded with the zinc phthalocyanine photosensitizer	hexagonal NaYF_4_ Yb^3+^/Er^3+^ (concentrations are not provided)	Exc. 980 nm emission in green (520–560 nm) and red (640–680 nm)	Effective against methicillin-resistant *S. aureus* (MRSA) and *E. coli*. Effective treatment of the MRSA-infected abscesses in deep tissue (1 cm).	[72]
Rose Bengal (photosensitizer)-loaded LiYF_4_ capped with polyvinylpyrrolidone	LiYF_4_ Yb^3+^/Er^3+^ (concentrations are not provided)	Exc. 980 nm (power density of 1 W/cm^2^) Emission in green (520–560 nm) and red (640–680 nm)	Effective in deep tissue infections; used with methylene blue for aPDT. Decline of 4.72 log_10_ in viability of drug-resistant *Acinetobacter baumannii* at a dose of 50 μg mL^−1^ UCNPs-PVP-RB.	[73]
Rose Bengal (photosensitizer)-loaded NaYF_4_:Yb,Er@NaGdF_4_:Nd@SiO_2_ core–shell nanoparticles	NaYF_4_ Yb^3+^/Er^3+^ (18/2 mol%)	Exc. 980 nm (power density of 1 W/cm^2^); emission in green (520–560 nm) and red (640–680) nm	Effective against methicillin-resistant *S. aureus* (MRSA) and *E. coli*.	[74]
D-TiO_2_/Au@SiO_2_@Y_2_O_3_:Yb^3+^,Er^3+^ with an antibiotic drug Ampicillin sodium covalently linked to the nanoparticles by a (3- glycidyloxypropyl)trimethoxysilane monolayer linker	Y_2_O_3_ Yb^3+^/Er^3+^ (concentrations are not provided)	Exc. 980 nm (power density of 0.68 W/cm^2^); emission in green (520–560 nm) and red (640–680 nm)	Effective against methicillin-resistant *S. aureus* (MRSA) and *E. coli*.	[75]
NaErF_4_:Tm^3+^@NaYF_4_:Yb^3+^-Chlorin e6-Mn(CO)_5_Br@Silane	NaErF_4_:Tm^3+^ Er^3+^/Tm^3+^ (concentrations are not provided) NaYF_4_ Yb^3+^ (concentration is not provided)	Exc. 980 nm (power density of 1 W/cm^2^). Emission in red (660 nm)	At 150 μg/mL, the therapy results in inhibition of over 70% of *E. coli* and *S. aureus*, while at 200 μg/mL, it inhibits approximately 90% of both bacteria strains. Additionally, an anti-inflammatory effect is observed.	[65]
Heavy metal-free organic photosensitizer attached to the NaGdF_4_:Nd^3+^/Tm^3+^/Yb^3+^@NaGdF_4_ core–shell nanoparticles coated with a phospholipid bilayer	NaGdF_4_ Yb^3+^/Nd^3+^/Tm^3+^ (25/1/0.5 mol%)	Exc. 808 nm (power density of 140 mW/cm^2^ or 3.2 W/cm^2^). Emission in UV (340, and 360 nm) and blue (450 nm and 480 nm).	HeLa cells are efficiently destroyed via 808 nm laser irradiance of 140 mW/cm^2^ for 3 min (<30% cell viability) or via 3.2 W/cm^2^ for 6 min (<10% cell viability).	[76]
(CTAB-coated NaYF_4_:Yb/Tm)@ZnO	β-NaYF_4_ Yb^3+^/Tm^3+^ (18/5 mol%)	Exc. LED 970 nm (power of 12 mW/cm^2^). Emission in UVA (345 and 362 nm) and blue (451 nm and 475 nm)	CFU reduction of *S. aureus* WCH-SK2-SCV of 82.6% and *S. aureus* WCH-SK2 of 78.8% is demonstrated.	[77]

**Table 2:** Pr^3+^-activated materials showing 4f5d emission in UVC. A more comprehensive list is given in Refs. [90,91].

Host	Crystal Structure	Space Group	Main Emission Peaks (nm)	Reference	$\mathrm{Cutoff}\;\mathrm{Phonon}\;\mathrm{Energy}\;\hbar \omega_{max}$(cm^−1^) with [Reference]
Cs_2_NaYCl_6_	Cubic	*Fm*3*m*	263, 277, 301, 314	[92]	284 [93]
LaOI	Tetragonal	*P4nmm*	300	[94]	<430 [95] *
KCaF_3_	Hexagonal	*Pnma*	257	[96]	412 [97]
RbCaF_3_	Cubic	*Pm*-3*m*	261	[96]	486 [98]
CsCaF_3_	Cubic	*Pm*-3*m*	250, 273	[96]	449 [98]
LiLuF_4_	Tetragonal	*I*41/*amd*	223–281	[99]	445 [100]
CaSO_4_	Orthorhombic	*Amma*	223, 234, 250, 255	[101]	1185 [102]
Cs_2_NaYF_6_	Cubic	*Fm*-3*m*	250, 270	[103]	467 [93]
YBO_3_	Hexagonal	*P*6_3_/*m*	263, 275	[89]	1368 [104]
La_2_CaB_10_O_19_	Monoclinic	*C*2	279, 334	[105]	1493 [106]
LuPO_4_	Tetragonal	*I*41/*amd*	235, 246, 263, 274	[87]	1161 [107]
YPO_4_	Tetragonal	*I*41/*amd*	232, 244.5, 261.6, 271	[108]	1149 [107]
NaCaPO_4_	Orthorhombic	*Pn*21*a*	251, 261, 282	[109]	1080 [110]
K_3_Lu(PO_4_)_2_	Trigonal	*P*-3	253, 282, 315	[87,111]	1147 [112] **
Sr_3_(PO_4_)_2_	Trigonal	*R*3-*m*	231, 269	[113]	1072 [114]
Sr_3_Y(PO_4_)_3_	Cubic	*I*-43*d*	248, 278	[115]	1080 [116]
Ba_3_Y(PO_4_)_3_	Cubic	*I*-43*d*	250, 280	[115]	1044 [117]
Ca_9_Y(PO_4_)_7_	Trigonal	*R*-3*c*	240, 275	[118]	1125 [118]
LiLuSiO_4_	Orthorhombic	*Pnma*	268, 283, 316	[87]	980 [119] ***
Li_2_SrSiO_4_	Hexagonal	*P*3121	265, 315	[120]	884 [121]
LiY_9_(SiO_4_)_6_O_2_	Hexagonal	*P*6_3_*/m*	268	[122]	958 [123]
Lu_2_SiO_5_	Monoclinic	*C*2*/c*	275, 313	[124]	970 [125]
X2-Y_2_SiO_5_	Monoclinic	*C*2*/c*	270, 282, 308	[126]	971 [127]

* LaOI has a lower phonon energy than LaOBr due to the larger atomic mass of I than Br; ** phonon energy of Rb_3_Lu(PO_4_)_2_; *** phonon energy of LiYbSiO_4_.

**Table 3:** Literature data on bactericidal effects induced by Pr^3+^ UC UVC radiation.

UC Material	Emission	Excitation	Antimicrobial Effect	Ref.
β-NaYF_4_:Pr^3+^/Li^+^ and β-NaYF_4_: Pr^3+^/Li^+^@BiOCl composite (dopant concentrations are not provided)	UVC (253 nm, 259 nm, 284 nm)	444 nm	Antimicrobial effect of β-NaYF_4_:Pr^3+^/Li^+^ under 444 nm excitation demonstrated. With the β-NaYF_4_: Pr^3+^/Li^+^@BiOCl composite, the effect is significantly improved (visible light excitation ≥ 420 nm kills 99.99% of *E. coli* in 180 min—aPDT effect).	[153]
Lu_7_O_6_F_9_:Pr^3+^ (1 mol%)	UVC 260 nm	447 nm	Inactivation of *E. coli* implicated by the authors.	[154]
NaYF_4_:Pr^3+^/Yb^3+^ (2/10 mol%) LiYF_4_:Pr^3+^/Yb^3+^ (1/10 mol%)	UVC 275 nm	447 nm	Significant denaturation of a double strand DNA after exposure to 447 nm radiation for 20 to 40 min. Material’s additional functionality is luminescence imaging in the NIR-II spectral region.	[155]
Cs_2_NaYF_6_:Pr^3+^ (1 mol%)	UVC afterglow at 250 nm	X-ray	Following 16 min of X-ray irradiation, the sample is placed near to a plate containing a colony of the Gram-negative bacteria *Pseudomonas aeruginosa*. The viability of around 40% of bacteria is seen under the UVC afterglow of this material.	[103]
Y_2_SiO_5_:Pr^3+^ (1 mol%) Y_2_SiO_5_:Pr^3+^/Gd^3+^ (1/1 mol%) Y_2_SiO_5_:Pr^3+^/Gd^3+^/Li^+^ (1.2/1.2/7.2 mol%) Y_2_SiO_5_:Pr^3+^/Li^+^ (1.2/7.2 mol%)	UVC 280 nm, additional emission at 318 nm with samples containing Gd^3+^	“daylight” fluorescent lighting	Inactivation of *B. subtilis* spores on dry phosphor-coated surfaces (best results with Pr^3+^/Gd^3+^/Li^+^-doped material). Inhibition of *P. aeruginosa* biofilms grown on the coated surfaces.	[142]
Ca_2_SiO_4_:Pr^3+^ (dopant concentration is not provided)	UVC 247 nm	450 nm laser	Inactivation of *B. subtilis.*	[156]
Y_2_SiO_5_:Pr^3+^ composite film with polyvinyl alcohol (PVA) and sodium alginate (SA)	UVC 280 nm	455 nm and white LED	Inhibition of Gram-positive *S. aureus* and Gram-negative *E. coli Pseudomonas aeruginosa* bacteria.	[152]
Y_2_Si_2_O_7_:Pr^3+^/Tm^3+^/Yb^3+^ (1.2/0.5/5 mol%)	UVC (278 nm) + UVB (308 nm) + UVA (370 nm)	447 nm laser, 800 mW/cm^2^	The viability of planktonic cultures of *A. baumannii*, *S. aureus*, and *C. albicans.*	[157]
Li_2_SrSiO_4_:Pr^3+^ (dopant concentration is not provided)	Two broad peaks (265 nm and 320 nm).	450 nm laser, 1 W	Inactivation of *Bacillus subtilis*: after 300 s of irradiation, the mortality rate reached 90%, and after 600 s of irradiation, almost all bacteria died.	[120]
Li_2_CaGeO_4_:Pr^3+^ (1 mol%)	UVC+UVB (~240–330 nm)	450 nm laser, 1 W	Complete inactivation of *S. aureus* bacteria in 30 min.	[158]
Li_2_SrGeO_4_ (1 mol%)	UVC+UVB (~240–330 nm)	450 nm laser diode, 0.6 W	Inactivation of *Staphylococcus aureus*, *Salmonella enterica*, *Klebsiella pneumoniae*, and *Escherichia coli* in 40 to 80 min.	[159]

**Table 4:** SOC values for Pr^3+^ materials whose emissions are shown in Figure 10; calculated using Equation (3).

	LaPO_4_	YPO_4_	Lu_7_O_6_F_9_	YBO_3_	X2-Y_2_SiO_5_	Sr_3_(BO_3_)_2_	Lu_2_SiO_5_	β-Y_2_Si_2_O_7_
SOC	0.282	0.357	0.674	0.529	0.533	0.535	0.504	0.477

## Data Availability

The data presented in this study are available upon request from the corresponding author.
